# Microbiota Signals Suppress B Lymphopoiesis With Aging in Mice

**DOI:** 10.3389/fimmu.2021.767267

**Published:** 2021-10-19

**Authors:** Joseph R. Krambs, Darlene A. Monlish, Feng Gao, Laura G. Schuettpelz, Daniel C. Link

**Affiliations:** ^1^ Division of Oncology, Department of Medicine, Washington University School of Medicine, Saint Louis, MO, United States; ^2^ Department of Pediatrics, Washington University School of Medicine, Saint Louis, MO, United States; ^3^ Department of Surgery, Washington University School of Medicine, Saint Louis, MO, United States

**Keywords:** hematopoiesis, aging, microbiome, B lymphopoiesis, hematopoietic stem cell, myeloid skewing, inflammation, HSC transplantation

## Abstract

Aging is associated with significant changes in hematopoiesis that include a shift from lymphopoiesis to myelopoiesis and an expansion of phenotypic hematopoietic stem cells (HSCs) with impaired self-renewal capacity and myeloid-skewed lineage differentiation. Signals from commensal flora support basal myelopoiesis in young mice; however, their contribution to hematopoietic aging is largely unknown. Here, we characterize hematopoiesis in young and middle-aged mice housed under specific pathogen free (SPF) and germ-free (GF) conditions. The marked shift from lymphopoiesis to myelopoiesis that develops during aging of SPF mice is mostly abrogated in GF mice. Compared with aged SPF mice, there is a marked expansion of B lymphopoiesis in aged GF mice, which is evident at the earliest stages of B cell development. The expansion of phenotypic and functional HSCs that occurs with aging is similar in SPF and GF mice. However, HSCs from young GF mice have increased lymphoid lineage output, and the aging-associated expansion of myeloid-biased HSCs is significantly attenuated in GF mice. Consistent with these data, RNA expression profiling of phenotypic HSCs from aged GF mice show enrichment for non-myeloid biased HSCs. Surprisingly, the RNA expression profiling data also suggest that inflammatory signaling is increased in aged GF HSCs compared with aged SPF HSCs. Collectively, these data suggest that microbiota-related signals suppress B lymphopoiesis at multiple stages of development and contribute to the expansion of myeloid-biased HSCs that occurs with aging.

## Introduction

Aging is associated with significant changes in hematopoiesis which includes a shift from lymphopoiesis to myelopoiesis (1–4). A decline in lymphopoiesis is evident early in adulthood and progressively declines with aging (5). Prior studies show that there is a loss of the earliest lymphoid-restricted progenitors in aged mice, including decreases in lymphoid-primed multipotent progenitors (LMPPs) and common lymphoid progenitors (CLPs) (6–9). Decreased proliferation and cytokine responsiveness of pre-pro-B, and pro-B cells also has been reported (6, 7, 10). Finally, there is evidence that impaired lineage-specification of hematopoietic stem cells (HSCs) may contribute to the decrease in lymphopoiesis. Specifically, phenotypic HSCs expand with age, but they have reduced self-renewal capacity and display myeloid-lineage skewing (1, 11, 12).

There is evidence suggesting that inflammation may contribute to age-related changes in hematopoiesis (13–18). Expression of certain inflammatory cytokines increases with aging, including tumor necrosis factor-alpha (TNF-α) and interleukin-1β (IL1-β) (19, 20). This is relevant to aging, since chronic stimulation with IL1-β results in reduced HSC self-renewal and enhanced myeloid differentiation (21), and TNF-α signaling has been implicated in the reduced proliferation of aged pro-B cells and myeloid skewing of HSCs (22, 23). Increased toll-like receptor signaling also has been implicated in hematopoietic aging. Prolonged (4-6 week) treatment with the TLR4 ligand, lipopolysaccharide (LPS), is associated with an expansion of phenotypic HSCs with reduced repopulating and enhanced myeloid differentiation, reproducing some of the most prominent features of hematopoietic aging (24). Likewise, prolonged treatment with a TLR2 agonist results in an expansion of phenotypic HSCs but a loss of HSC self-renewal capacity (25).

One source of inflammatory signaling is the microbiota. Prior studies have established through the study of germ-free mice or antibiotic treated mice that signals from microbiota play an important role in the regulation of hematopoiesis (26, 27). A consistent finding from multiple groups is modestly reduced myelopoiesis in germ-free or antibiotic treated young mice, with decreases in mature neutrophils, monocytes, and myeloid progenitors (27–29). The impact of microbiota on multipotent hematopoietic progenitors is less clear, with two groups showing a modest reduction in phenotypic HSCs and multipotent progenitors in germ-free or antibiotic treated mice (26, 30), while our data using young germ free mice showed no difference in HSC number or quiescence (31). In the present study, we examine the impact of microbiota on basal hematopoiesis in young adult or middle aged mice. Our data suggest that microbiota signals play an important role in the age-dependent shift from lymphopoiesis to myelopoiesis.

## Methods

### Mice and Mouse Housing

All mouse experiments were approved by Washington University Animal Studies Committee. Germ-free C57BL/6J mice were housed in a sterile environment of plastic flexible film isolators, as described previously (32). Animal feces and swabs of the insides surfaces of the isolator were monitored on a weekly basis for microbial contamination. Specific pathogen-free mice were maintained under specific pathogen-free conditions. All experiments were done using young 6-8-week-old mice and aged 10-12-month-old mice. Equal numbers of each gender were used. Additional details on mouse strain and housing are provided in **Supplementary Methods**.

### Flow Cytometry and Cell Sorting

Bone marrow and peripheral blood were processed for flow cytometry as previously described (33). Data were acquired using a FACS Aria III flow cytometer and analyzed using FlowJo™ v10.6.1 software. Cell sorting was performed using the FACS Aria III flow cytometer cell sorter. The LSK-SLAM population was double sorted for purity, improving purity to greater than 90%. A complete list of antibody clones used in these experiments is provided in **Supplementary Methods**.

### Transplantation

Six- to eight-week-old wild-type *Ly5.1/Ly5.2* recipient mice were irradiated twice with 600 cGy 6 hours apart. Donor (Ly5.2) bone marrow cells were then injected retro-orbitally and placed on prophylactic antibiotics (trimethoprim-sulfamethoxazole) for 2 weeks. Peripheral blood chimerism was analyzed every 4 weeks until mice were sacrificed 24 weeks after transplantation when donor chimerism in bone marrow and blood were analyzed. For the sorted HSPC transplantation experiments, 50 lineage^-^ Sca1^+^ cKit^+^ CD150^+^ CD48^-^ (LSK-SLAM) cells were double sorted into single wells of a 96 well plate containing 250,000 support whole (Ly5.1) bone marrow cells and then injected into recipient mice. HSC purity of the double sorted was greater than 90%. Only mice with at least 1% trilineage donor chimerism were used to assess lineage output.

### RNA Expression Profiling

Libraries were generated from RNA purified from sorted LSK-SLAM was hybridized to Agilent SurePrint G3 Mouse G3 arrays. Details on library preparation, hybridization and analysis of the RNA expression profiling data are provided in **Supplementary Methods**. RNA expression data is available in the Gene Expression Omnibus database (GSE183138).

### Serum Inflammatory Mediator Measurement

The level of 40 different cytokines, chemokines, or acute phase proteins in the serum of mice was quantified using the Mouse Cytokine Antibody Array, Panel A, as per manufacturer’s recommendations (R&D systems, Minneapolis, MN).

### Statistical Analysis

For single parameter analysis, unpaired t-test were used to assess statistical significance. For multiple parameter data, statistical significance was calculated using one-way (ANOVA). For the limiting dilution analysis, a log-fraction plot of the limiting dilution model was fitted to data from limiting dilution transplantation of 25K, 50K, or 100K cells from whole bone marrow preparations. The log-fraction plots and statistical analysis were generated using the Extreme Limiting Dilution Analysis software (34).

## Results

### Microbiota Signals Contribute to the Suppression of B Lymphopoiesis With Aging

**Figure 1:**
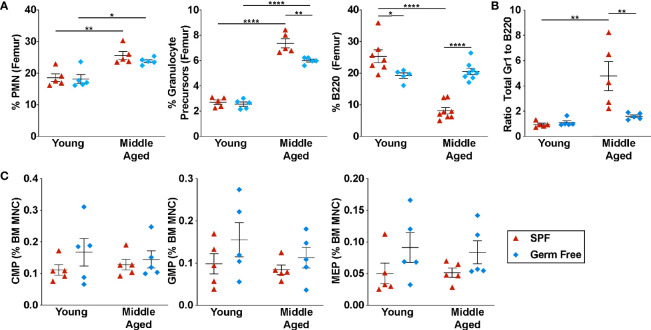
Microbiota signals contribute to the suppression of B lymphopoiesis. **(A)** Percentage of mature neutrophils (Gr1^hi^ SSC^hi^ cells, granulocyte precursors (CD115^+^ Gr1^Int^ cells), and B220^+^ cells in the bone marrow. **(B)** Ratio of Gr1^+^ cells to B220^+^ cells in the bone marrow. **(C)** Percentage of common myeloid progenitors (CMP, lineage^–^ Sca1^–^ Kit^+^ CD34^+^ CD16/32^–^ cells), granulocyte-macrophage progenitors (GMP, lineage^–^ Sca1^–^ Kit^+^ CD34^+^ CD16/32^+^ cells), and megakaryocyte-erythrocyte progenitors (MEP, lineage^–^ Sca1^–^ Kit^+^ CD34^–^ CD16/32^–^ cells) in the bone marrow. Middle aged is defined as 10-12 months. Data represent the mean ± SEM. *P < 0.05, **P < 0.01, and ****P < 0.0001 by one-way ANOVA with alpha = 0.05 and Sidak’s multiple comparisons test. Technical replicates were completed at least three times over the span of four years due to availability of germ-free mice.

To investigate the impact of the microbiome on hematopoiesis during aging, we analyzed young (6-8 weeks) and middle-aged (~12 months) mice (hereafter referred to as aged mice) housed under specific pathogen free (SPF) or GF conditions. We did not analyze older mice due to the difficulty in maintaining mice in a gnotobiotic facility for more than one year. However, age-associated changes in hematopoiesis, including myeloid skewing, lymphoid progenitor reduction, and reduced self-renewal capacity are evident in C57BL/6 mice by 12 months (6, 9, 35). Complete blood counts were similar between SPF and GF mice in both young and aged mice, except for a mild anemia in aged SPF mice (**Supplementary Figure 1A**). As reported previously (27–29), the percentage of and absolute number of circulating neutrophils (Gr1^hi^ CD115^–^ SSC^hi^ cells) and monocytes (CD115^+^ Gr1^low/neg^ cells) is reduced in young GF mice (**Figure 1A** and **Supplementary Figures 1B, C**). Interestingly, this difference is lost in aged GF mice. In the bone marrow of young GF mice, the percentage and absolute number of neutrophils, Gr1-intermediate granulocytic precursors (CD115^+^ Gr1^Int^ cells), and myeloid progenitors are normal, suggesting that granulopoiesis is intact (**Figures 1A, C**, **Supplementary Figures 2B–D**).

Consistent with prior studies, there is a shift in hematopoiesis in aged SPF mice towards granulopoiesis, with a significant increase in the percentage of granulocytic cells and a decrease in B lineage cells in the bone marrow (**Figure 1A**) (6, 36, 37). Due to the increase in BM cellularity in aged mice (**Supplementary Figure 2A**), on an absolute basis, there is a marked increase in granulocytic cells, while B lineage cell number is unchanged (**Supplementary Figure 2B**). This shift from B-lymphopoiesis to granulopoiesis with aging is mostly abrogated in GF mice. Compared with aged SPF mice, a significant increase in the percentage of B lineage cells was observed in the bone marrow and blood of aged GF mice, with a modestly attenuated increase in granulocytic cells (**Figures 1A, B** and **Supplementary Figure 2B**). Indeed, whereas the ratio of granulocytic cells to B lineage cells in the bone marrow of SPF mice increased nearly 4-fold with aging, no increase was observed in GF mice (**Figure 1B**).

### Microbiota Signals Regulate B Lymphopoiesis at Multiple Stages of Development

**Figure 2:**
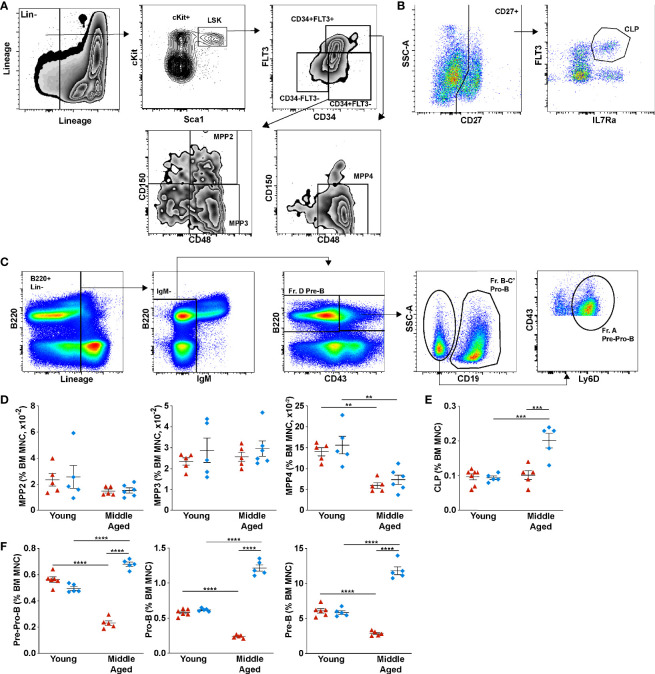
Microbiota signals suppress B lymphopoiesis through down-regulating common lymphoid progenitors in aged mice. **(A)** Representative flow plots of young SPF mouse BM showing the gating strategy used to identify multipotent progenitor (MPP) populations MPP2 (lineage^–^ Sca1^+^ Kit^+^ CD34^+^ FLT3^–^ CD48^+^ CD150^+^), MPP3 (lineage^–^ Sca1^+^ Kit^+^ CD34^+^ FLT3^–^ CD48^+^ CD150^–^), and MPP4 (lineage^–^ Sca1^+^ Kit^+^ CD34^+^ FLT3^+^ CD48^+^ CD150^–^). **(B)** Common lymphoid progenitors (lineage^–^ CD27^+^ FLT3^+^ IL7Rα^+^ cells) **(C)** B cell progenitors Pre-Pro-B (lineage^–^ B220^+^ IgM^–^ CD19^–^ CD43^+^ Ly6D^+^ cells), Pro-B (lineage^–^ B220^+^ IgM^–^ CD19^+^ CD43^+^ cells), and Pre-B (lineage^–^ B220^+^ IgM^–^ CD43^–^ cells). **(D)** Percent of multipotent MPP2, MPP3, and MPP4 per femur. **(E)** Percentage of common lymphoid progenitors **(F)** Percentage of Pre-Pro-B cells, Pro-B cells, Pre-B cells and Immature B cells (lineage^–^ B220^+^ IgD^–^ IgM^+^ cells) in the bone marrow. Data represent the mean ± SEM. **P < 0.01, ***P < 0.001, and ****P < 0.0001 by one-way ANOVA with alpha = 0.05 and Sidak’s multiple comparisons test.

We next examined B lymphopoiesis, quantifying different stages of B cell development starting with lymphoid-primed multipotent progenitors (LMPP/MMP4, lineage^–^ Sca1^+^ Kit^+^ CD34^+^ FLT3^+^ CD48^+^ CD150^–^), lymphoid-committed common lymphoid progenitors (CLPs, lineage^–^ CD27^+^ FLT3^+^ IL7Rα^+^ cells), and the following B cell precursors: pre-pro-B cells (lineage^–^ B220^+^ IgM^–^ CD19^–^ CD43^+^ Ly6D^+^ cells), pro-B cells (lineage^–^ B220^+^ IgM^–^ CD19^+^ CD43^+^ cells), and pre-B (lineage^–^ B220^+^ IgM^–^ CD43^–^ cells). Representative flow plots showing the gating strategy to identify each cell population are shown in **Figures 2A–C**; gating was based on young SPF mice. Consistent with a recent study, a decrease in the percentage of MMP4s, but not MMP2s or myeloid-primed MMP3s was observed in middle aged SPF mice (**Figure 2D**) (9). A similar trend was observed in middle aged GF mice. Although no change in the percentage of CLPs were observed, a significant decrease in most B cell precursors populations was observed in middle aged SPF mice (**Figures 2E, F**). This trend was reversed in GF mice, with significant increases in the percentage and absolute number of CLPs and B cell precursors induced with aging (**Supplementary Figure 3**). These data suggest the microbiota signals contribute to the suppression of B lymphopoiesis during aging primarily at the CLP stage.

### Microbiota Signals Are Not Required for the Aging-Dependent Increase in HSPCs

**Figure 3:**
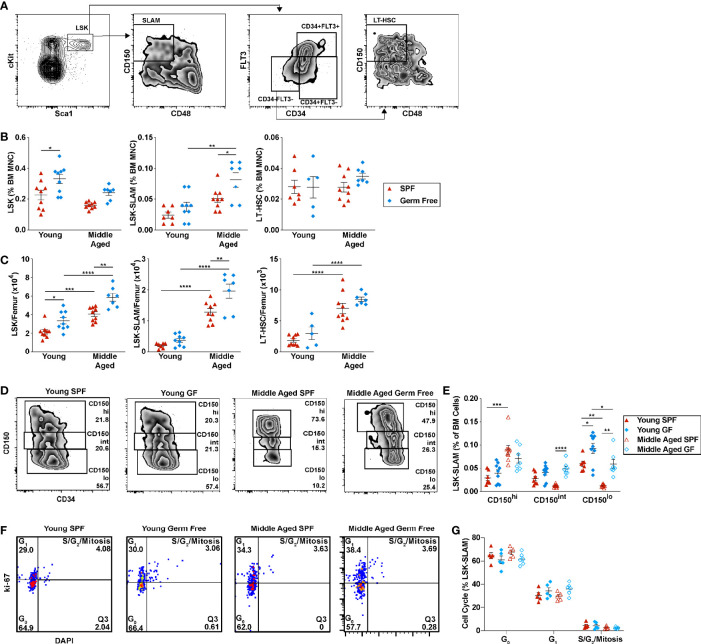
Microbiota signals are not required for the expansion of HSCs with aging. **(A)** Representative flow plots of young SPF mouse BM showing the gating strategy used to identify LSK, LSK-SLAM, and LSK-SLAM CD34- (LT-HSC) cells. **(B)** Percent and **(C)** number of LSK, LSK-SLAM, and LSK-SLAM CD34^-^ cells (LT-HSC) per femur. **(D)** Representative flow plots showing the frequency of CD150^hi^, CD150^int^, and CD150^lo^ HSCs; data are gated on LSK cells. CD150 gating for each experiment was adjusted based on FMO controls. **(E)** Frequency of CD150^hi^, CD150^int^, and CD150^lo^ HSCs. **(F)** Representative flow plots showing the gating strategy used to identify cell cycle status of LSK-SLAM cells. **(G)** Cell cycle distribution of LSK-SLAM cells. Data represent the mean ± SEM. *P < 0.05, **P < 0.01, ***P < 0.001, and ****P < 0.0001 by one-way ANOVA with alpha = 0.05 and Sidak’s multiple comparisons test.

The impact of microbiota signals on HSCs, especially with aging, is not well characterized. To address this issue, we first quantified HSCs by flow cytometry (**Figures 3A–C**). On a percentage basis, a non-significant trend to increased phenotypic HSCs was observed in SPF mice with aging (**Figure 3B**). On an absolute basis, the increase in phenotypic HSCs is highly significant (**Figure 3C**). In particular, the number of lineage^-^ Sca1^+^ cKit^+^ CD150^+^ CD48^-^ (LSK-SLAM) cells and CD34^-^ LSK-SLAM cells is increased 6.4 ± 1.7-fold and 3.4 ± 1.2-fold, respectively. Similar increases were observed in aged GF mice, with LSK-SLAM increasing 5.3 ± 1.6-fold (p=NS compared to SPF mice) and CD34^-^ LSK-SLAM cells increasing 2.8 ± 0.31-fold (p=NS). Aging is associated with an increase in myeloid biased HSCs (38). Prior studies have shown that high CD150 expression marks myeloid biased HSCs (9). Thus, we next assessed CD150 expression on phenotypic HSCs (LSK, CD34^-^ cells). As expected, a significant increase in the percentage of CD150-high HSCs and a corresponding decrease in CD150-low HSCs was observed in aged SPF mice (**Figures 3D, E**). This shift was largely attenuated in GF mice. Indeed, a significant increase in CD150-low lymphoid biased HSCs is present in both young and aged GF mice compared with aged-matched SPF mice. Of note, no significant difference in the cell cycle status of lineage- Kit+ committed progenitors or LSK-SLAM cells was observed with aging in either SPF or GF mice (**Figures 3F, G** and **Supplementary Figure 4**).

**Figure 4:**
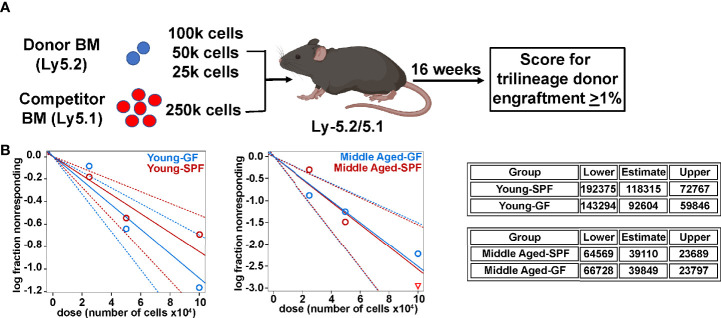
Microbiota signals do not alter age related expansion of HSCs. **(A)** Limiting dilution transplantation strategy to measure frequency of functional HSCs. Created with BioRender.com. **(B)** Limiting dilution analysis of repopulating activity. Shown is the fraction of mice showing long-term multilineage engraftment versus dose of total bone marrow cells transplanted. The dotted lines give the 95% confidence interval. The table represents exact one-sided 95% confidence interval estimates of HSC frequency.

Prior studies have shown that phenotypic HSCs from aged mice have reduced repopulating activity on a per cell basis (2, 39, 40). Thus, we performed limiting dilution transplantation using unsorted bone marrow cells as an unbiased approach to assess functional HSC frequency. As reported previously (4), the number of functional HSCs increases in the bone marrow of SPF with age (**Figures 4A, B**). A similar expansion of functional HSCs is present in GF mice. Together, these data suggest that signals from the microbiota are dispensable for the expansion of phenotypic and functional HSCs that occurs with chronologic aging.

### Microbiota Signals Contribute the Lineage Bias of HSCs

**Figure 5:**
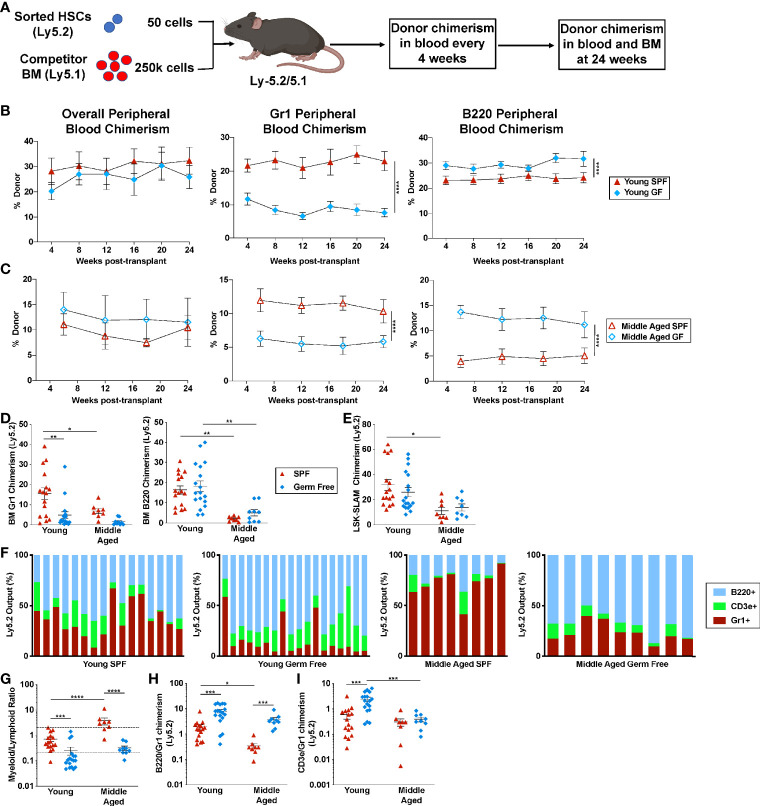
Microbiota signals suppress the lymphoid potential of aged HSCs. **(A)** Experimental schema. Created with BioRender.com. **(B)** Fifty sorted LSK-SLAM cells from the indicated donors were transplanted into irradiated wildtype SPF mice and donor peripheral blood chimerism assessed every four to six weeks. Shown is donor (Ly5.2) contribution to total leukocytes, granulocytes and B cells in recipients of transplanted young HSCs **(B)** or aged HSCs **(C)**. **(D, E)** Donor chimerism in bone marrow Gr-1^+^ cells and B220^+^ cells **(D)** or LSK-SLAM cells **(E)** harvested 24 weeks after transplantation. **(F)** Shown is the contribution of each lineage to the total donor cell pool; each bar indicates an individual recipient. **(G)** Quotient of donor bone marrow myeloid cells (Gr1^+^) cells to lymphoid (CD3e^+^ plus B220^+^) cells for each recipient. Myeloid-biased mice had a ratio > 2, lineage balanced were between 0.25 and 2, and lymphoid-biased mice had a ratio <0.25. **(H, I)** Ratio of donor bone marrow B220^+^ to Gr1^+^ chimerism **(H)** or donor bone marrow CD3e^+^ to Gr1^+^ chimerism (**I**). Data represent the mean ± SEM. *P < 0.05, **P < 0.01, ***P < 0.001, and ****P < 0.0001 by one-way ANOVA with alpha = 0.05 and Sidak’s multiple comparisons test.

To assess the impact of microbiota on HSC lineage commitment, we transplanted a limiting number of sorted HSCs and assessed lineage output (**Figure 5A**). Stable overall donor engraftment was observed over time, with reduced engraftment of aged HSCs from both SPF and GF mice (**Figures 5B, C, E**). A significant decrease in donor granulocytic cell chimerism was seen in both young and aged GF HSC recipients (**Figures 5B–D**). Conversely, donor B cell chimerism in the blood was increased in aged GF HSC recipients, with a similar trend observed in the bone marrow 24 weeks after transplantation. We next analyzed individual mice with at least 1% trilineage engraftment 24 weeks after transplantation to assess HSC lineage bias. As expected, in young SPF mice, the majority of HSCs displayed a balanced myeloid/lymphoid lineage output, with a significant increase in myeloid-biased HSCs observed with aging (**Figures 5F, G**). In young GF mice, the majority of HSCs are lymphoid-biased. Moreover, although the myeloid output, as measured by granulocytic cell chimerism, increased modestly with aging, the majority of HSCs in aged GF remained lymphoid-biased or balanced. In young GF HSCs, an increase in the output of both B- and T-lineage cells was observed (**Figures 5H, I**). In contrast in aged GF HSCs, the increase in lymphoid output was mainly due to an increased production of B cells. Collectively, these data show that microbiota-related signals play a key role in determining the lineage potential of bone marrow resident HSCs, promoting myeloid lineage development at the expense of lymphoid cells.

**Figure 6:**
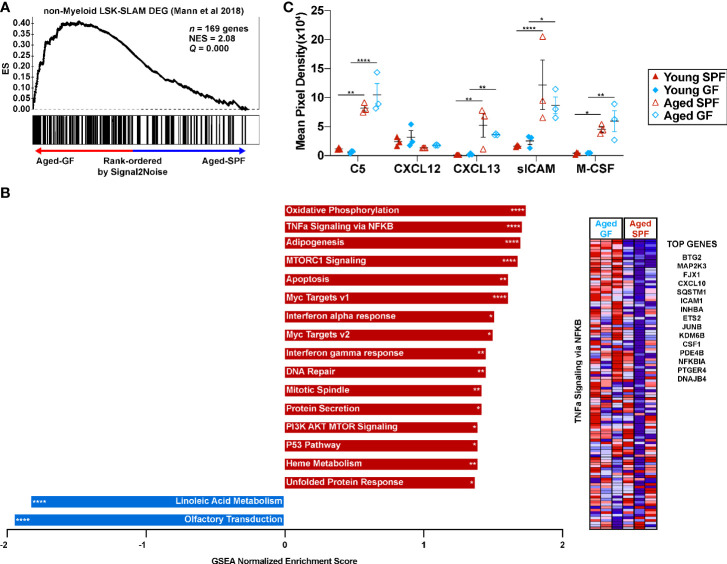
Microbiota signals enhance the myeloid gene signature of HSCs with age but are not required for expression of inflammatory mediators in the blood. **(A)** Gene set enrichment analysis for genes consistent with non-myeloid biased LSK-SLAM cells. **(B)** Significantly enriched gene sets from the Hallmark, KEGG, and Reactome gene sets (adjusted FDR< 0.1, nominal p-value<0.05). Red bars represent gene sets upregulated in aged GF HSCs and blue bars gene sets upregulated in aged SPF HSCs. *P < 0.05, **P < 0.01, ***P < 0.001, and ****P < 0.0001 by GSEA. Heatmap of the TNFa signaling *via* NFKB gene set with a list of the top 15 differentially expressed genes between aged GF and aged SPF. **(C)** Quantification of arrays that interrogate inflammatory cytokines/chemokines hybridized with serum. Data represent the mean ± SEM. *P < 0.05, **P < 0.01, and ****P < 0.0001 by two-way ANOVA with alpha = 0.05 and Sidak’s multiple comparisons test.

To begin to explore mechanisms by which microbiota signals regulate HSC lineage bias, gene expression profiling was performed on sorted LSK-SLAM cells from aged SPF and aged GF mice. A list of differentially expressed genes is provided in **Supplementary Table 1**. Consistent with our lineage-output transplantation data, aged GF HSCs had a gene expression signature enriched for non-myeloid biased HSCs (**Figure 6A** and **Supplementary Figure 5**) (41). Surprisingly, gene set enrichment analysis showed that GF HSCs were enriched for expression signatures related to inflammatory signaling, including tumor necrosis factor (TNF) and interferon (IFN) signaling (**Figure 6B**). This prompted us to measure the circulating level of 40 different inflammatory cytokines, chemokines, or acute phase proteins, including TNFα, interleukin-1β, and IFN gamma. Only five of these inflammatory mediators were detected above background (**Figure 6C**). Increased expression of C5, CXCL13, soluble ICAM, and M-CSF was observed in the serum of SPF mice with aging, with a similar increase seen in aged GF mice. Together, these data suggest the microbiota signals are not major drivers of increased systemic inflammatory cytokine/chemokine expression or inflammatory signaling in HSCs.

## Discussion

Prior studies suggest that aging impacts B lymphopoiesis at multiple stages of development. First, HSCs become myeloid-based with aging (1, 3, 4, 12). Second, Young et al. provided evidence that the lymphoid potential of MMP4/LMPP decreases with aging (9). Finally, several studies have shown that there is a loss of early B-cell committed progenitors (6–9). Our data are consistent with these observations, although most prior studies have reported a decrease in CLPs in aged (18-24 month old) mice. This discrepancy may be due to the younger age (12-14 month) of SPF mice analyzed in our study. Indeed, Young et al. showed that CLPs progressively decline with age and are within normal limits in 12 month old mice (9). Our data suggest the microbiota signals contribute to the suppression of B lymphopoiesis with aging primarily by regulating the production and/or maintenance of committed B cell progenitors, although molecular mechanisms are unclear. Of note, prior studies suggest that reduced interleukin-7 responsiveness (6), or increased senescence (7) or TNFα-induced apoptosis (22) may contribute to the loss of B cell precursors with aging. Whether any of these mechanisms are dependent on microbiota will require further study.

Our data suggest that microbiota signals play an important role in determining the lineage bias of HSCs.

Consistent with prior studies (1–4), we show that aging is associated with an expansion of phenotypic HSCs with reduced repopulating activity in mice. Despite the decrease in repopulating activity on a per cell basis, the large increase in phenotypic HSCs with aging compensates for this loss. Indeed, limiting dilution studies using total bone marrow cells show that the number of long-term repopulating HSCs is actually increased in aged mice (4). This is consistent with recent single cell RNA sequencing studies suggesting that there is marked expansion of myeloid-restricted HSCs, along with a moderate expansion of multipotent HSCs (41, 42). Interestingly, the number of phenotypic and functional HSCs increases to a similar degree with aging in GF mice, suggesting that microbiome signals do not contribute to this phenotype. A consistent finding in many studies, including our own, is an increase in myeloid-biased HSCs with aging (1, 3, 4, 10–12). Our transplantation studies show that the lymphoid lineage output of young GF HSCs is increased compared with SPF HSCs. With aging, although there is some shift towards increased myeloid cell production, lymphoid cell production, particularly B lineage cell production, remains high in GF HSCs. Consistent with this conclusion, a recent paper showed that aged GF mice maintain HSCs with balanced lympho-myeloid lineage output upon transplantation (43). There are some caveats to the transplantation studies, including the use of irradiation in recipient mice which induces transient systemic inflammation (44, 45) and the short-term treatment of recipients with antibiotics, which may alter the microbiota. Although these factors were controlled for in our experimental approach, the data suggest that microbiota result in, as yet undefined, epigenetic changes in HSCs that contribute to lineage specification.

The mechanisms regulating lineage bias of HSCs is not well understood. Numerous prior studies comparing young to aged HSCs have identified alterations in the epigenome, metabolism, cell polarity, and proteostasis (reviewed in Mejia-Ramirez et al.) (46). Reactive oxygen species (ROS) levels increase in aged HSCs and correlate with myeloid lineage skewing and reduced long-term repopulating activity (47). Although we did not measure ROS, our RNA expression profiling suggest that aged GF HSCs are more metabolically active, with increased oxidative phosphorylation compared to aged SPF HSCs. There is considerable interest in the role of inflammation in HSC aging. Chronic stimulation with certain inflammatory cytokines or TLR ligands results in an HSC aging phenotype, with a loss of HSC repopulating and enhanced myeloid differentiation (21, 24, 25). Moreover, He et al. recently provided evidence that elevated TNFα signaling, by increasing IL27RA expression in HSCs, contributes to their biased myeloid differentiation (23). Most recently, Kovtonyuk et al. provided compelling data suggesting that interleukin-1 contributes to the increase in myeloid biased HSC with aging. Of note, they showed that blocking interleukin-1 signaling or suppression of gut microbiota with oral antibiotics was able to partially revert the myeloid-biased HSC aging phenotype. Surprisingly, our RNA expression profiling data suggest that inflammatory signaling (including TNFα and interferon) is increased in aged GF HSCs compared to SPF HSCs. However, the relationship between the microbiota and inflammation is likely to be complex and further study of the mechanisms by which microbiota and inflammatory signaling regulate HSC lineage output is needed.

In summary, these data show that microbiota signals contribute to hematopoietic aging and HSC lineage specification. The nature of the signals remains an open and important question, whose answer will provide important new insights into the regulation of hematopoiesis.

## Data Availability Statement

The datasets presented in this study can be found in online repositories. The names of the repository/repositories and accession number(s) can be found in the article/**Supplementary Material**.

## Ethics Statement

The animal study was reviewed and approved by Washington University in St. Louis Institutional Animal Care and Use Committee.

## Author Contributions

DL and LS conceived and jointly supervised the study. JK, LS, and DL designed the experiments. JK, DM, and LS performed the experiments. JK and DL wrote the manuscript. JK and FG performed statistical analyses. All authors contributed to the article and approved the submitted version.

## Funding

The Siteman Cancer Center is supported in part by an NCI Cancer Center Support Grant #P30 CA091842. This study was supported by the National Institutes of Health grants R01HL131655 (DL), R01HL134896 (LS) and F31CA247136 (JRK), and the Children’s Discovery Institute of Washington University and St. Louis Children’s Hospital (LS).

## Conflict of Interest

The authors declare that the research was conducted in the absence of any commercial or financial relationships that could be construed as a potential conflict of interest.

## Publisher’s Note

All claims expressed in this article are solely those of the authors and do not necessarily represent those of their affiliated organizations, or those of the publisher, the editors and the reviewers. Any product that may be evaluated in this article, or claim that may be made by its manufacturer, is not guaranteed or endorsed by the publisher.
