# Supplementary material for: Microbiota Signals Suppress B Lymphopoiesis With Aging in Mice
Source: Front Immunol. 2021 Oct 19;12:767267. doi: 10.3389/fimmu.2021.767267 (PMC8560790; doi:10.3389/fimmu.2021.767267)
Supplement: Supplementary file 1 [file DataSheet_1.pdf]

SUPPLEMENTAL MATERIALS AND METHODS

Mice and Animal Housing

All mouse experiments were performed using Washington University Animal Studies Committee approved protocols. Germ-free C57BL/6J mice were housed in the sterile environment of plastic flexible film isolators (Class Biologically Clean Ltd., Madison, WI), as described previously.[1] Manipulations are carried out within the sterile workspace using neoprene gloves directly attached to the bag by metal compression rings. A double-door port is located in the wall of the isolator opposite the gloves. This port is used to bring supplies into the isolator. Air is supplied to each isolator by a dedicated blower attached to a filter by a flexible vinyl hose. Air exits the isolator through an identical filter assembly. Air pressure within the isolator is kept slightly above atmospheric, so that the isolator remains inflated and immediate entrance of airborne contaminants is prevented in the case of accidental puncture. Sterile supplies, such as autoclaved food, water, and bedding, are brought into the isolator through the side port. Since germ-free mice lack nutrients synthesized by gut microbes (e.g. vitamin K), mice were maintained on an enriched rodent diet (BeeKay autoclavable rodent diet; B&K Universal).

Routine monitoring of isolators for the presence of bacteria and fungi is imperative when maintaining a germ-free colony. Animal feces were monitored on a weekly basis for microbial contamination. In addition, the inside surfaces of the isolator were swabbed and cultured. All samples were cultured aerobically and anaerobically in three different media: nutrient broth (a general-purpose medium for cultivating microorganisms with non-exacting nutritional requirements), brain/heart infusion broth (allows cultivation of a wide variety of fastidious microorganisms), and Sabouraud Dextrose Broth (supports growth of yeasts, molds, and aciduric microorganisms). All three media are available from BD Difco. Anaerobic incubations are done at 37°C in GasPak jars (Fisher) with activated GasPak Hydrogen + CO_2 envelopes (Fisher). Aerobic incubations are done at 37°C and 42°C.

Specific pathogen-free mice were maintained under standard pathogen-free conditions and include C57BL/6 (Ly45.2 wild-type) and B6.SJL-*Ptprc^{a} Pepc^{b}*/BoyJ (Ly45.1 wild-type) mice which were obtained from The Jackson Laboratory (Bar Harbor, ME). Mice were crossed in-house to generate Ly5.2/Ly5.1 expressing mice on a C57BL/6 background. All experiments were done using young 6-8-week-old mice and aged 10-12-month-old mice. Transplant donor and support/competitor mice were age and sex matched. Recipient mice were 6-8 weeks old.

Flow cytometry and cell sorting

Peripheral blood, bone marrow, and spleen mononuclear cell (MNC) preparations were red blood cells lysed. The resulting cell suspension was filtered through CellTrics 70µm nylon filters (Sysmex, Goerlitz, Germany) to generate single cell suspensions. Mononuclear cells (MNCs) were counted using trypan blue (Thermo Fisher, Waltham, MA) and a Cellometer Auto T4 Bright Field Cell Counter (Nexcelom, Lawrence, MA). Complete blood counts were obtained using the HV950 hemavet (Drew Scientific, Miami Lakes, FL). MNCs were incubated with target antibodies at 4°C for 30 minutes in phosphate buffered saline (PBS) containing 1mM ethylenediaminetetraacetic acid (EDTA) and 0.2% (weight/volume) bovine serum albumin (BSA). All antibodies were obtained from BioLegend (San Diego, CA). Data were acquired using a FACS Aria III flow cytometer (BD biosciences, San Jose, CA) and analyzed using FlowJo™ v10.6.1 software (BD biosciences). LSK-SLAM cells were sorted using a Sony iCyt Synergy SY3200 (Synergy) cell sorter (Sony, San Jose, CA).

The hematopoietic stem/progenitor cell (HSPC) panel included PE-Cy7-conjugated anti-CD117 (2B8); PerCP-Cy5.5-conjugated anti-Ly-6A/E; PE-conjugated anti-CD150 (TC15-12F12.2) BV421-conjugated anti-CD48 (HM48-1); APC-conjugated anti-CD16/32 (2.4G2); FITC-conjugated anti-CD34 (RAM34); and BV711-conjugated anti-CD135 (A2F10.1) and the following APC-Cy7-conjugated antibodies: anti-CD3e (145-2C11), anti-B220 (RA3-6B2), anti-Gr1 (RB6-8C5), anti-Ter119 (TER-119), anti-CD11b (M1/70), and anti-NK1.1 (PK136). The B progenitor panel included FITC-conjugated anti-Ly6D (49-H4), PE-conjugated anti-CD43 (S11), PE-Cy7-conjugated anti-CD19 (6D5), APC-conjugated anti-IgM (RMM1), BV421-conjugated anti-B220 (RA3-6B2), PerCP-Cy5.5-conjugated anti-IgD (11-26c.2a), and the following APC-Cy7-conjugated antibodies: anti-CD11c (N418), anti-CD3e (145-2C11), and anti-NK1.1 (PK136). The common lymphoid progenitor panel included BV421-conjugated anti-IL7Rα (A7R34), PerCP-Cy5.5-conjugated anti-CD27 (LG.3A10), APC-conjugated anti-CD135 (A2F10), FITC-conjugated anti-Ly6D (49-H4), and the following APC-Cy7-conjugated antibodies: anti-CD3e (145-2C11), anti-B220 (RA3-6B2), anti-Gr1 (RB6-8C5), anti-Ter119 (TER-119), anti-CD11b (M1/70), anti-CD11c (N418), and anti-NK1.1 (PK136). Antibodies were obtained from BioLegend or BD Biosciences.

RNA expression profiling

RNA was purified from sorted KSL-SLAM cells using the Qiagen RNeasy Micro Kit (74004, Qiagen). Following amplification of RNA using the WTA2 kit (Sigma Aldrich, St. Louis, MO), libraries were generated using the Kreatech ULS RNA labeling kit (Leica Biosystems, Wetzlar, Germany) and hybridized to the Agilent SurePrint G3 Mouse GE microarray (Santa Clara, CA), containing 62,976 probes for 24,241 annotated genes. Raw images were first passed-through quality control tools from the Bioconductor package of R[2]. Array intensity data were then background adjusted, and quantile normalized[3]. To test for differential expression, the linear models for Microarray data (limma) package was used[4]. Features were filtered based on normalized expression >100 within all groups and coefficient of variation ≤75 within all groups. Gene set enrichment analysis was performed using the GSEA v4.0.3 software. Enrichment score (ES) and False discovery rate (FDR) value were applied to filter pathways enriched after gene set permutations were performed 1000 times for the analysis.

Supplementary References

1. Hooper LV, Mills, J.C., Roth, K.A., Stappenbeck, T.S., Wong, M.H. & Gordon, J.I. Combining gnotobiotic mouse models with functional genomics to define the impact of the microflora on host physiology. *Methods in Microbiology.* 2002;31:559-589.
2. Huber W, Carey VJ, Gentleman R, et al. Orchestrating high-throughput genomic analysis with Bioconductor. *Nat Methods.* 2015;12(2):115-121.
3. Bolstad BM, Irizarry RA, Astrand M, Speed TP. A comparison of normalization methods for high density oligonucleotide array data based on variance and bias. *Bioinformatics.* 2003;19(2):185-193.
4. Ritchie ME, Phipson B, Wu D, et al. limma powers differential expression analyses for RNA-sequencing and microarray studies. *Nucleic Acids Res.* 2015;43(7):e47.

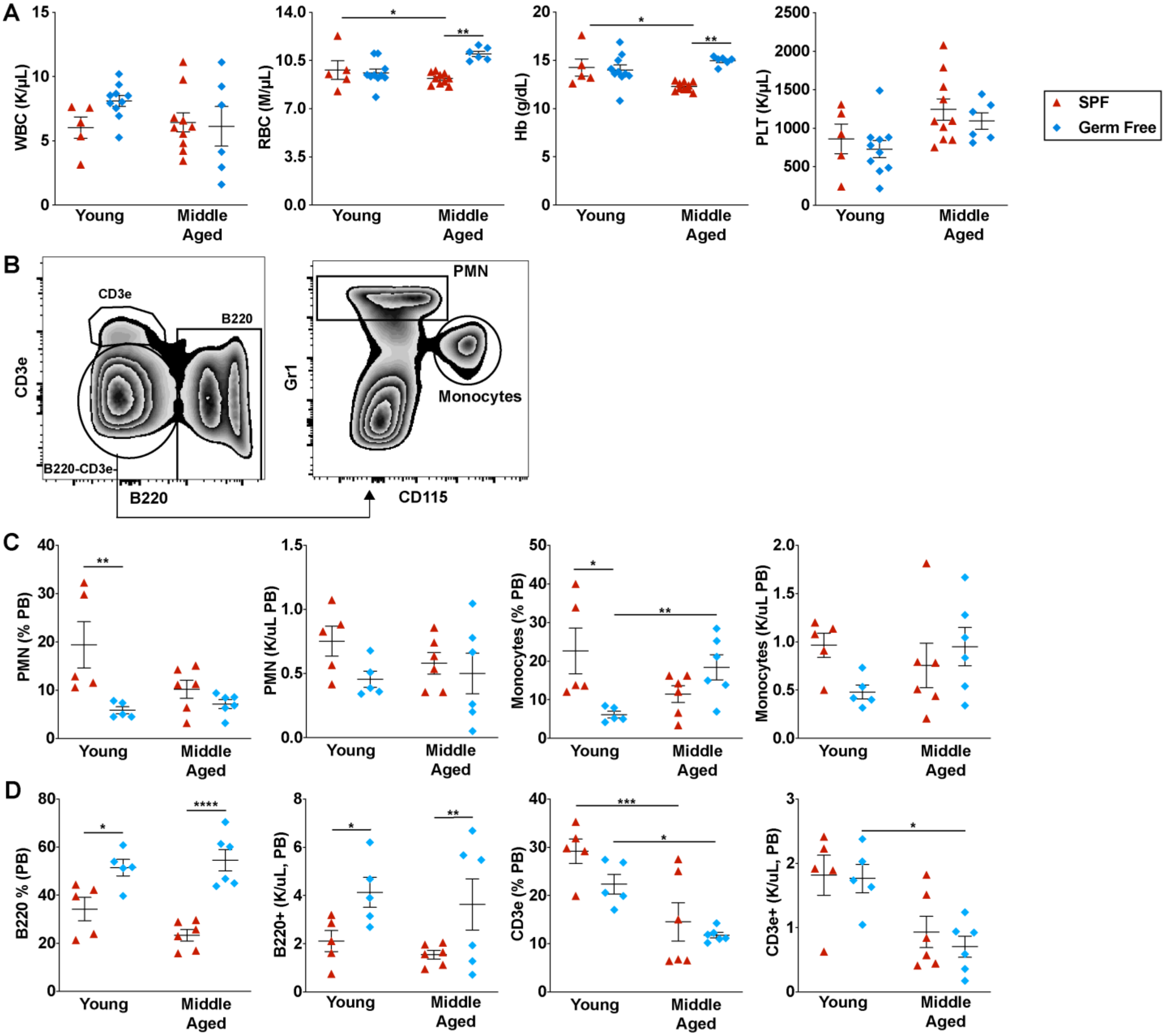

Supplementary Figure 1. Basal hematopoiesis in young and aged GF mice. (**A**) Peripheral blood counts. (**B**) Representative flow plots showing gating strategy to identify B220+ (B cells), CD3e+ (T cells), Gr1hi SSC-hi CD115- (neutrophils), and Gr1int CD115+ (monocytes) cells in the blood. (**C**) Number and percent of circulating neutrophils (PMNs, $Gr1^{hi}$ $CD115^{-}$ SSC^{hi} cells) and monocytes ($CD115^{+}$ $Gr1^{low/neg}$ cells). (**D**) Number and percent of circulating $CD3\varepsilon^{+}$ cells (T-lineage) and $B220^{+}$ cells (B-lineage). Data represent the mean ± SEM. *P < 0.05, **P < 0.01, ***P < 0.001, and ****P < 0.0001 by one-way ANOVA with alpha = 0.05 and Sidak's multiple comparisons test.

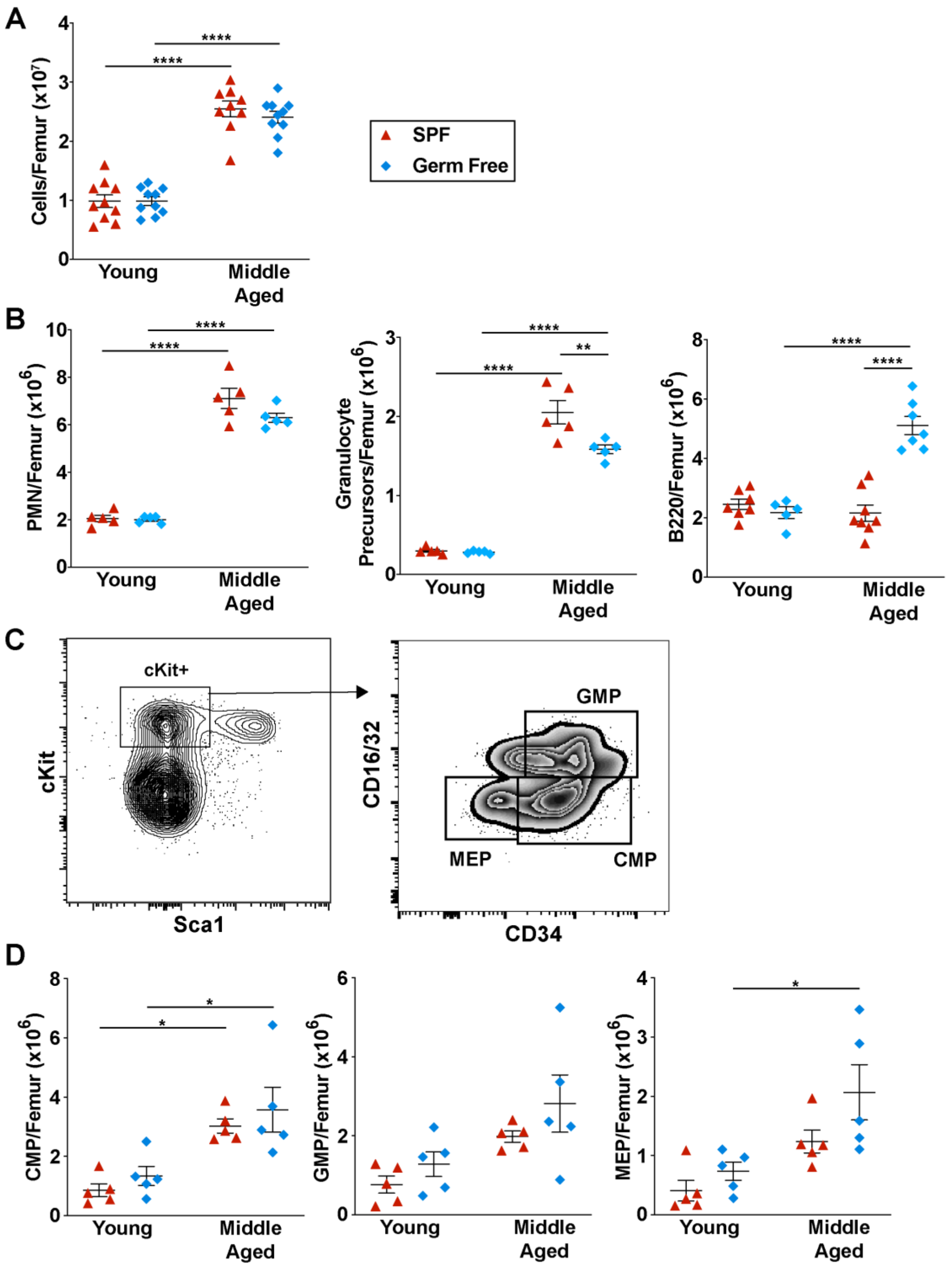

Supplementary Figure 2. Basal hematopoiesis in young and aged GF mice. (A) Bone marrow cellularity. **(B)** Number of B220$^+$, CD115$^+$ Gr1^{Int} cells and mature neutrophils (Gr1^{hi} SSC^{hi} cells) in the bone marrow. (**C**) Representative flow plot gating of common myeloid progenitors (CMP, lineage$^-$ Sca1$^-$ Kit$^+$ CD34$^+$ CD16/32$^-$ cells), granulocyte-macrophage progenitors (GMP, lineage$^-$ Sca1$^-$ Kit$^+$ CD34$^+$ CD16/32$^+$ cells), and megakaryocyte-erythrocyte progenitors (MEP, lineage$^-$ Sca1$^-$ Kit$^+$ CD34$^-$ CD16/32$^-$ cells) in the bone marrow. (**D**) Number of CMP, GMP, and MEP per femur. Data represent the mean ± SEM. *P < 0.05, **P < 0.01, ***P < 0.001, and ****P < 0.0001 by one-way ANOVA with alpha = 0.05 and Sidak's multiple comparisons test.

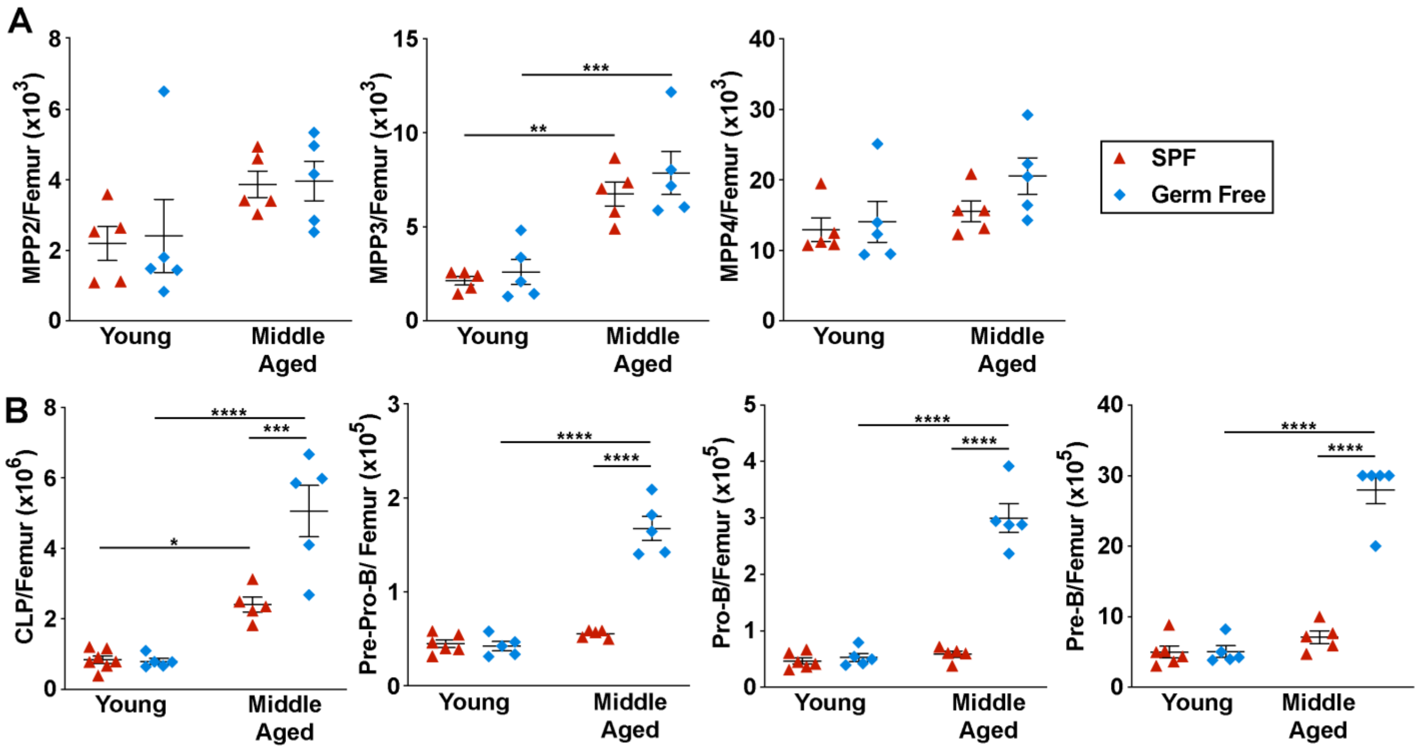

Supplementary Figure 3. Basal hematopoiesis in young and aged GF mice. (A) Number of multipotent progenitor 2 (MPP2, lineage$^-$ Sca1$^+$ Kit$^+$ CD34$^+$ FLT3$^-$ CD48$^+$ CD150$^+$), MPP3 (lineage$^-$ Sca1$^+$ Kit$^+$ CD34$^+$ FLT3$^-$ CD48$^+$ CD150$^-$), and MPP4 (lineage$^-$ Sca1$^+$ Kit$^+$ CD34$^+$ FLT3$^+$ CD48$^+$ CD150$^-$) per femur. (**B**) Number of common lymphoid progenitors (CLP, lineage$^-$ CD27$^+$ FLT3$^+$ IL7Rα^+ cells), Pre-Pro-B cells (lineage$^-$ B220$^+$ IgD$^-$ IgM$^-$ CD19$^-$ CD43$^+$ Ly6D$^+$ cells), Pro-B cells (lineage$^-$ B220$^+$ IgD$^-$ IgM$^-$ CD19$^+$ CD43$^+$ cells), and Pre-B cells (lineage$^-$ B220$^+$ IgD$^-$ IgM$^-$ CD19$^+$ CD43$^-$ cells) in the bone marrow. Data represent the mean ± SEM. *P < 0.05, **P < 0.01, ***P < 0.001, and ****P < 0.0001 by one-way ANOVA with alpha = 0.05 and Sidak's multiple comparisons test.

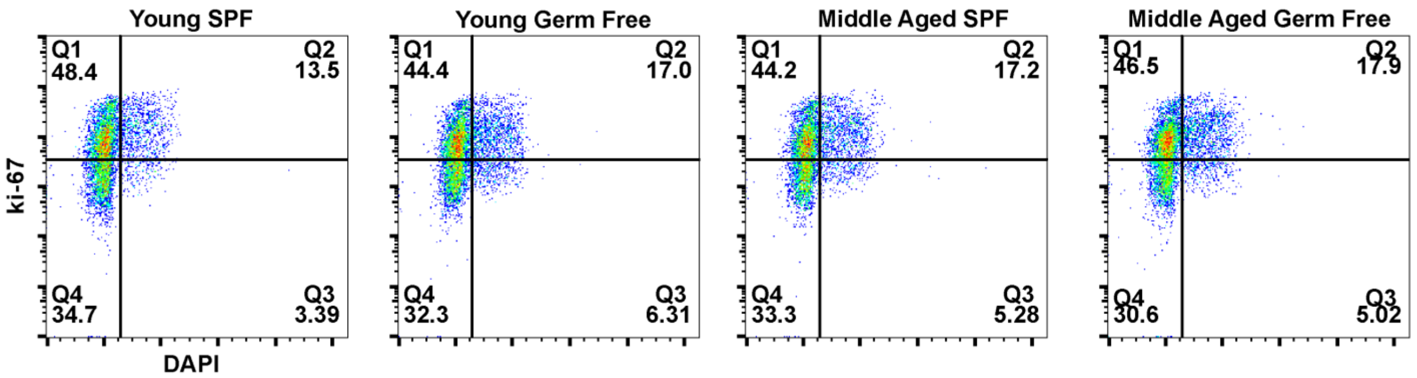

Supplementary Figure 4. Cell cycle status of Kit$_{+}$ lineage^{-} hematopoietic progenitors. Representative flow plots showing DAPI and Ki-67 staining of Kit^{+} lineage^{-} cells harvested from the bone marrow of the indicated mice. Cells in the G_0, G_1, G_2/S/M phase of the cell cycle are located in Q4, Q1, and Q2, respectively.

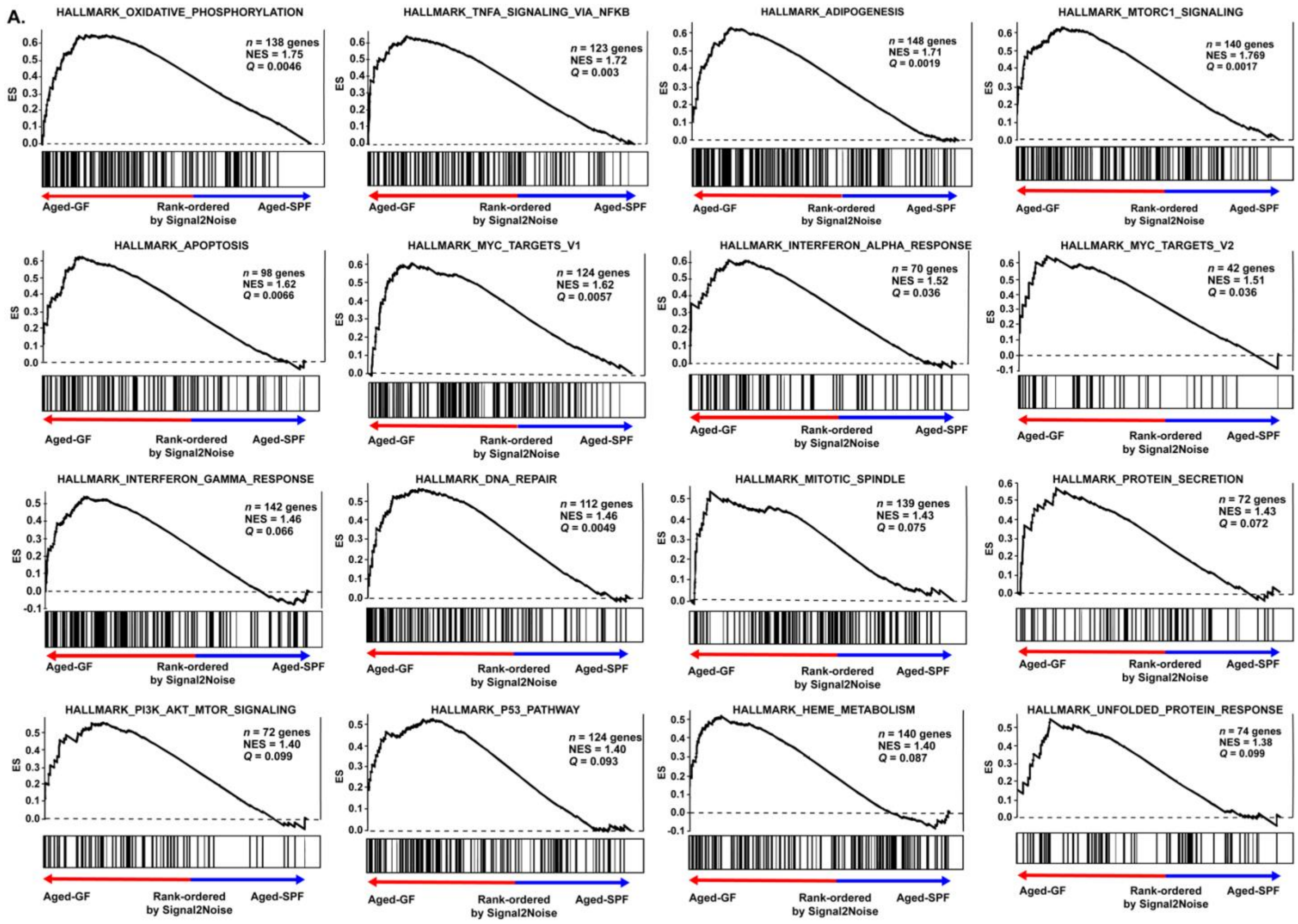

Supplementary Figure 5. Gene set enrichment analysis of aged GF and SPF HSCs RNA expression data.

(**A**) Significantly enriched gene sets from the Hallmark, KEGG, and Reactome gene sets (adjusted FDR< 0.1, nominal p-value<0.05, and have >25 genes).

Supplementary Table 1. Differentially expressed genes in hematopoietic stem cells of aged GF compared to aged SPF mice.

Gene Symbol	Aged SPF1	Aged SPF2	Aged SPF3	Aged GF1	Aged GF2	Aged GF3	Aged SPF Average	Aged GF Average	Fold change (Aged GF vs. Aged SPF)	FDR
HIF1AN	167	194	24	182	654	1059	128.33	631.67	4.92	3.25E-03
CANT1	451	182	299	1252	651	2639	310.67	1514	4.87	5.49E-04
RASD1	141	103	80	614	252	607	108	491	4.55	6.13E-04
IGF2	154	159	153	933	660	522	155.33	705	4.54	1.50E-04
TTYH2	402	460	456	2081	763	2520	439.33	1788	4.07	2.46E-04
ISLR	261	280	49	824	613	891	196.67	776	3.95	7.27E-04
INHBA	257	180	58	946	414	579	165	646.33	3.92	1.15E-03
TMEM132A	345	59	176	474	401	1245	193.33	706.67	3.66	3.88E-03
CXCL10	539	166	272	980	1534	825	325.67	1113	3.42	1.17E-03
VMA21	214	115	168	403	636	643	165.67	560.67	3.38	5.87E-04
2010107G12RIK	169	81	57	480	252	269	102.33	333.67	3.26	5.13E-03
GM10560	192	232	97	343	418	927	173.67	562.67	3.24	6.61E-03
AK048000	336	404	261	822	771	1634	333.67	1075.67	3.22	1.12E-03
NOL10	371	438	93	1587	623	627	300.67	945.67	3.15	4.09E-03
HTRA1	134	71	197	313	371	574	134	419.33	3.13	6.55E-03
MYO1H	159	361	118	647	378	956	212.67	660.33	3.11	4.42E-03
GM21057	140	95	91	329	369	313	108.67	337	3.1	2.60E-03
F630042J09RIK	173	192	91	410	379	624	152	471	3.1	8.82E-03
BTG2	191	187	261	524	561	873	213	652.67	3.06	3.00E-03
AW045869	212	401	269	1387	646	624	294	885.67	3.01	3.00E-03
TSPAN1	218	236	48	548	381	563	167.33	497.33	2.97	5.88E-03
ZFP689	338	413	603	1614	713	1587	451.33	1304.67	2.89	1.91E-03
H2-AA	11564	14066	6915	29263	36490	27910	10848.33	31221	2.88	8.84E-04
AK187431	295	196	390	569	655	1293	293.67	839	2.86	2.39E-03
SAMM50	1457	1564	1107	4399	3069	3955	1376	3807.67	2.77	8.65E-05
GM32819	208	384	93	428	501	970	228.33	633	2.77	7.53E-03
HRAS	1209	875	917	2023	1380	4890	1000.33	2764.33	2.76	3.61E-03
DHRS3	9239	7616	15451	31406	35081	19306	10768.67	28597.67	2.66	1.96E-04
TLR12	900	681	399	1251	1241	2780	660	1757.33	2.66	6.90E-03
B3GNT8	658	644	272	1083	1015	2050	524.67	1382.67	2.64	3.74E-03
PKLR	221	154	197	401	394	673	190.67	489.33	2.57	9.71E-03
BF536591	573	303	546	569	1369	1655	474	1197.67	2.53	9.12E-03
LDLRAD1	101	211	49	377	288	250	120.33	305	2.53	9.41E-03
UCK1	1297	837	1176	3507	1646	3127	1103.33	2760	2.5	1.52E-03
LTB	7995	2706	3314	7329	7580	20138	4671.67	11682.33	2.5	4.27E-03

CSL	479	159	330	871	553	984	322.67	802.67	2.49	4.93E-03
PPP4C	2356	2009	1438	2329	3900	8193	1934.33	4807.33	2.49	6.93E-03
B3GNT7	139	207	77	384	316	352	141	350.67	2.49	8.02E-03
COQ5	425	233	210	689	569	858	289.33	705.33	2.44	4.05E-03
ZFP873	258	142	236	541	340	667	212	516	2.43	8.20E-03
GGA1	933	658	532	2048	830	2231	707.67	1703	2.41	9.23E-03
SLC25A10	741	416	864	2148	1447	1220	673.67	1605	2.38	2.03E-03
KCNA3	366	550	701	1475	1012	1296	539	1261	2.34	1.42E-03
TBC1D22BOS	737	1582	2416	5132	2861	3030	1578.33	3674.33	2.33	3.78E-03
CLNS1A	1186	614	939	2825	1138	2381	913	2114.67	2.32	4.85E-03
CD74	57489	29744	25031	86962	84070	87755	37421.33	86262.33	2.31	1.77E-04
PPIE	2381	3015	1899	4437	5067	7274	2431.67	5592.67	2.3	4.85E-04
MED22	2672	1773	2252	4266	3749	7385	2232.33	5133.33	2.3	1.05E-03
PIGH	329	450	698	1473	708	1221	492.33	1134	2.3	4.57E-03
ADRB2	503	864	542	1881	1651	838	636.33	1456.67	2.29	5.88E-03
D86604	473	884	783	1615	1346	1900	713.33	1620.33	2.27	2.50E-03
TRAF3IP2	571	1045	983	2779	1546	1529	866.33	1951.33	2.25	3.72E-03
KCNK5	416	502	309	953	667	1138	409	919.33	2.25	6.79E-03
NENF	808	803	1320	2604	1383	2548	977	2178.33	2.23	5.60E-03
ARPC4	20094	20785	15067	55573	30585	38160	18648.67	41439.33	2.22	5.29E-04
CLEC7A	1214	1337	1622	3818	2130	3302	1391	3083.33	2.22	1.81E-03
NAPSA	14218	10883	7868	27652	17546	27537	10989.67	24245	2.21	1.31E-03
RABGGTB	375	678	371	1003	1463	675	474.67	1047	2.21	6.41E-03
PIM3	1366	714	718	1519	1482	3135	932.67	2045.33	2.19	6.05E-03
RPRL2	77591	41797	54770	154110	90812	135610	58052.67	126844	2.18	3.16E-03
1700122E12RIK	6075	9383	9852	19179	17350	18303	8436.67	18277.33	2.17	2.03E-04
GEMIN4	2335	1493	1493	4635	3157	3708	1773.67	3833.33	2.16	8.71E-04
IER5	890	376	801	1151	1526	1778	689	1485	2.16	4.10E-03
CGRRF1	461	736	656	1491	1160	1338	617.67	1329.67	2.15	2.96E-03
CLDN10	489	822	1233	2594	1604	1271	848	1823	2.15	5.72E-03
RPRL3	57853	42122	44186	126423	69369	111362	48053.67	102384.67	2.13	3.87E-03
TNFRSF12A	939	957	971	1923	1547	2574	955.67	2014.67	2.11	2.15E-03
H2-DMB2	1352	793	806	1908	2320	1978	983.67	2068.67	2.1	4.69E-03
GBP9	4110	4470	2432	8814	8660	5466	3670.67	7646.67	2.08	2.35E-03
CSF1	3335	1185	1564	4433	3014	5189	2028	4212	2.08	5.15E-03
MBLAC1	603	574	1138	1620	1115	2028	771.67	1587.67	2.06	7.40E-03
SHMT1	1330	833	1296	3115	1426	2597	1153	2379.33	2.06	9.41E-03
GM5779	21035	35576	31188	59228	53717	66603	29266.33	59849.33	2.04	2.24E-04
PSMB5	21167	15617	15651	27639	27091	52086	17478.33	35605.33	2.04	3.66E-03
RPP30	593	866	1420	2131	2009	1727	959.67	1955.67	2.04	4.01E-03
COA5	817	543	715	1267	1563	1392	691.67	1407.33	2.03	3.11E-03
CLP1	1564	2280	3301	5845	3231	5395	2381.67	4823.67	2.03	5.95E-03

PYGB	4915	3529	3483	6538	6439	11082	3975.67	8019.67	2.02	4.39E-03
OAZ1	23122	20230	14003	21333	39504	54911	19118.33	38582.67	2.02	5.47E-03
BANF1	7875	6899	9301	10100	12573	25865	8025	16179.33	2.02	9.08E-03
NAB2	5749	4674	4335	8827	7409	13477	4919.33	9904.33	2.01	2.52E-03
H2-AB1	28364	24920	24009	52123	52674	50509	25764.33	51768.67	2.01	5.03E-03
ORAOV1	18181	12230	7065	21806	22718	30892	12492	25138.67	2.01	5.89E-03
SPATS2L	2941	2781	1798	1296	1450	1011	2506.67	1252.33	-2	3.55E-03
IL2RB	1019	915	964	396	747	289	966	477.33	-2.02	7.39E-03
COL15A1	1112	1225	2119	531	927	742	1485.33	733.33	-2.03	9.68E-03
AK084079	1243	1184	913	540	706	375	1113.33	540.33	-2.06	8.79E-03
OLFR537-PS1	1322	1741	1703	510	886	902	1588.67	766	-2.07	2.91E-03
BQ416348	2412	4874	2947	1355	1855	1679	3411	1629.67	-2.09	5.44E-03
4930511M06RIK	61169	135367	106587	32142	73827	39196	101041	48388.33	-2.09	7.01E-03
FDXACB1	23188	37430	23196	11871	16799	11426	27938	13365.33	-2.09	7.14E-03
TULP2	1901	1136	1057	503	793	649	1364.67	648.33	-2.1	5.75E-03
4921518K17RIK	713	1093	730	498	366	336	845.33	400	-2.11	7.79E-03
4930509J09RIK	1007	971	1148	384	646	443	1042	491	-2.12	6.35E-03
ELF5	1010	893	687	501	225	498	863.33	408	-2.12	7.22E-03
VAX2	23031	59989	35074	17309	24341	13705	39364.67	18451.67	-2.13	8.41E-03
4933409G03RIK	822	915	724	329	527	287	820.33	381	-2.15	4.83E-03
BRWD3	75584	124053	80942	26043	69134	35480	93526.33	43552.33	-2.15	6.02E-03
GM11213	1705	1054	970	396	686	650	1243	577.33	-2.15	6.35E-03
FPR2	1051	2374	1854	956	1061	433	1759.67	816.67	-2.15	7.95E-03
4930512M02RIK	177825	255879	262471	99748	162502	60498	232058.3	107582.67	-2.16	5.30E-03
AK037291	558	621	509	329	279	173	562.67	260.33	-2.16	9.11E-03
AK031035	806	792	1082	422	501	307	893.33	410	-2.18	6.29E-03
PHOX2A	940	2034	1622	748	738	627	1532	704.33	-2.18	7.35E-03
GM11944	1159	1182	1269	354	638	657	1203.33	549.67	-2.19	3.80E-03
AK029233	3641	1575	2219	671	792	1913	2478.33	1125.33	-2.2	8.25E-03
ADRA2B	115617	271897	141447	65850	123615	50378	176320.3	79947.67	-2.21	2.85E-03
ADAM25	660	1469	1445	377	711	524	1191.33	537.33	-2.22	9.64E-03
GM5953	1053	953	1280	495	534	440	1095.33	489.67	-2.24	2.20E-03
GM2090	3005	2849	2617	1473	1189	1090	2823.67	1250.67	-2.26	3.02E-03
KIRREL3	2065	4240	1863	1528	1229	864	2722.67	1207	-2.26	5.89E-03
OLFR1360	1080	826	696	310	388	441	867.33	379.67	-2.28	5.13E-03
HHLA1	1635	3377	1650	740	1304	867	2220.67	970.33	-2.29	3.58E-03
GM26911	465	813	550	338	345	116	609.33	266.33	-2.29	9.46E-03
9630013D21RIK	5995	5001	6937	2467	3154	2169	5977.67	2596.67	-2.3	1.26E-03
GM12128	1219	959	1067	389	825	194	1081.67	469.33	-2.3	4.46E-03
GM5833	26122	8388	10628	4842	5742	9033	15046	6539	-2.3	9.40E-03
GM4787	1745	1925	908	860	689	398	1526	649	-2.35	6.70E-03

BM934693	1014	1905	647	368	820	320	1188.67	502.67	-2.36	9.83E-03
GM11482	884	2417	1161	464	886	526	1487.33	625.33	-2.38	8.24E-03
GM36551	1268	3538	1513	719	1120	821	2106.33	886.67	-2.38	8.32E-03
OLFR103	744	623	897	423	406	120	754.67	316.33	-2.39	9.41E-03
HCN4	395	477	407	94	301	137	426.33	177.33	-2.4	7.30E-03
RAB9B	1330	786	808	309	476	429	974.67	404.67	-2.41	3.64E-03
ANKLE1	8860	25055	12244	5774	7318	5880	15386.33	6324	-2.43	4.59E-03
GM6042	527	725	896	267	321	293	716	293.67	-2.44	9.09E-03
GM32093	2898	1949	1425	900	1014	635	2090.67	849.67	-2.46	1.43E-03
GM15482	1162	892	653	199	641	259	902.33	366.33	-2.46	6.56E-03
2210407C18RIK	605	477	408	181	228	191	496.67	200	-2.48	5.86E-03
DLX6	2454	1086	1214	514	735	658	1584.67	635.67	-2.49	7.93E-03
OLFR554	410	812	779	251	225	320	667	265.33	-2.51	4.60E-03
TNXB	589	625	473	180	256	231	562.33	222.33	-2.53	3.04E-03
FSTL4	451	479	403	179	234	113	444.33	175.33	-2.53	9.21E-03
OLFR722	1248	459	766	389	260	318	824.33	322.33	-2.56	8.21E-03
CNGA3	560	1333	1013	377	598	156	968.67	377	-2.57	4.59E-03
B230303O12RIK	651	1294	1131	470	571	153	1025.33	398	-2.58	6.32E-03
ZFP366	8757	6590	5504	2461	3963	1542	6950.33	2655.33	-2.62	2.50E-04
GM6116	2990	3589	1952	1044	1441	774	2843.67	1086.33	-2.62	3.89E-04
OLFR46	1466	1599	2037	502	680	758	1700.67	646.67	-2.63	7.70E-04
CES4A	476	500	620	111	281	214	532	202	-2.63	4.30E-03
GM32975	604	367	719	124	301	209	563.33	211.33	-2.67	3.81E-03
OLFR773	1467	876	1018	639	556	61	1120.33	418.67	-2.68	9.21E-03
BY550418	406	904	831	275	376	138	713.67	263	-2.71	4.00E-03
GM32930	797	279	734	166	354	142	603.33	220.67	-2.73	8.11E-03
SLC35E3	468	638	320	255	160	105	475.33	173.33	-2.74	3.30E-03
AK031296	1861	945	623	529	364	318	1143	403.67	-2.83	3.96E-03
GPR61	1033	767	515	135	170	496	771.67	267	-2.89	6.68E-03
ARID3C	548	1646	1799	233	374	764	1331	457	-2.91	4.11E-03
ZFP385B	529	418	294	120	195	112	413.67	142.33	-2.91	6.97E-03
APOB	490	474	515	230	202	70	493	167.33	-2.95	6.87E-03
BC052181	607	598	846	394	119	178	683.67	230.33	-2.97	8.68E-04
ZFP750	222	529	666	235	203	27	472.33	155	-3.05	7.98E-03
GM6999	306	399	374	183	137	31	359.67	117	-3.07	4.01E-03
STARD6	592	306	234	142	196	27	377.33	121.67	-3.1	9.46E-03
OLFR1451	461	278	429	88	135	127	389.33	116.67	-3.34	2.46E-03
MMP12	899	598	992	55	374	247	829.67	225.33	-3.68	3.52E-03
PRP2	481	401	347	154	77	97	409.67	109.33	-3.75	6.86E-04
KCNH7	1474	673	310	327	67	232	819	208.67	-3.92	6.83E-03
4930417H01RIK	586	575	372	133	153	48	511	111.33	-4.59	5.16E-04
AK034071	1146	1534	942	148	441	180	1207.33	256.33	-4.71	2.44E-05

GHRH	582	881	641	141	244	37	701.33	140.67	-4.99	1.97E-04
GM10714	1317	369	597	29	143	159	761	110.33	-6.9	1.66E-04

Shown are normalized expression values from SurePrint G3 Mouse GE 8x60K Microarray calculated with limma. The average expression level of each gene for both conditions are calculated and shown as Aged SPF Average or Aged GF Average. Fold change is calculated as (Aged GF Average/Aged SPF Average). FDR, false discovery rate.
